# Supplementary material for: CRISPR-Cas9 target-strand nicking provides phage resistance by inhibiting replication
Source: bioRxiv. 2024 Sep 5:2024.09.05.611540. Preprint. [Version 1] doi: 10.1101/2024.09.05.611540 (PMC11398490; doi:10.1101/2024.09.05.611540)
Supplement: Supplement 2 [file NIHPP2024.09.05.611540v1-supplement-2.pdf]

bioRxiv preprint doi: https://doi.org/10.1101/2024.09.05.611540; this version posted September 5, 2024. The copyright holder for this preprint (which was not certified by peer review) is the author/funder, who has granted bioRxiv a license to display the preprint in perpetuity. It is made available under aCC-BY 4.0 International license.

Supplementary Figures

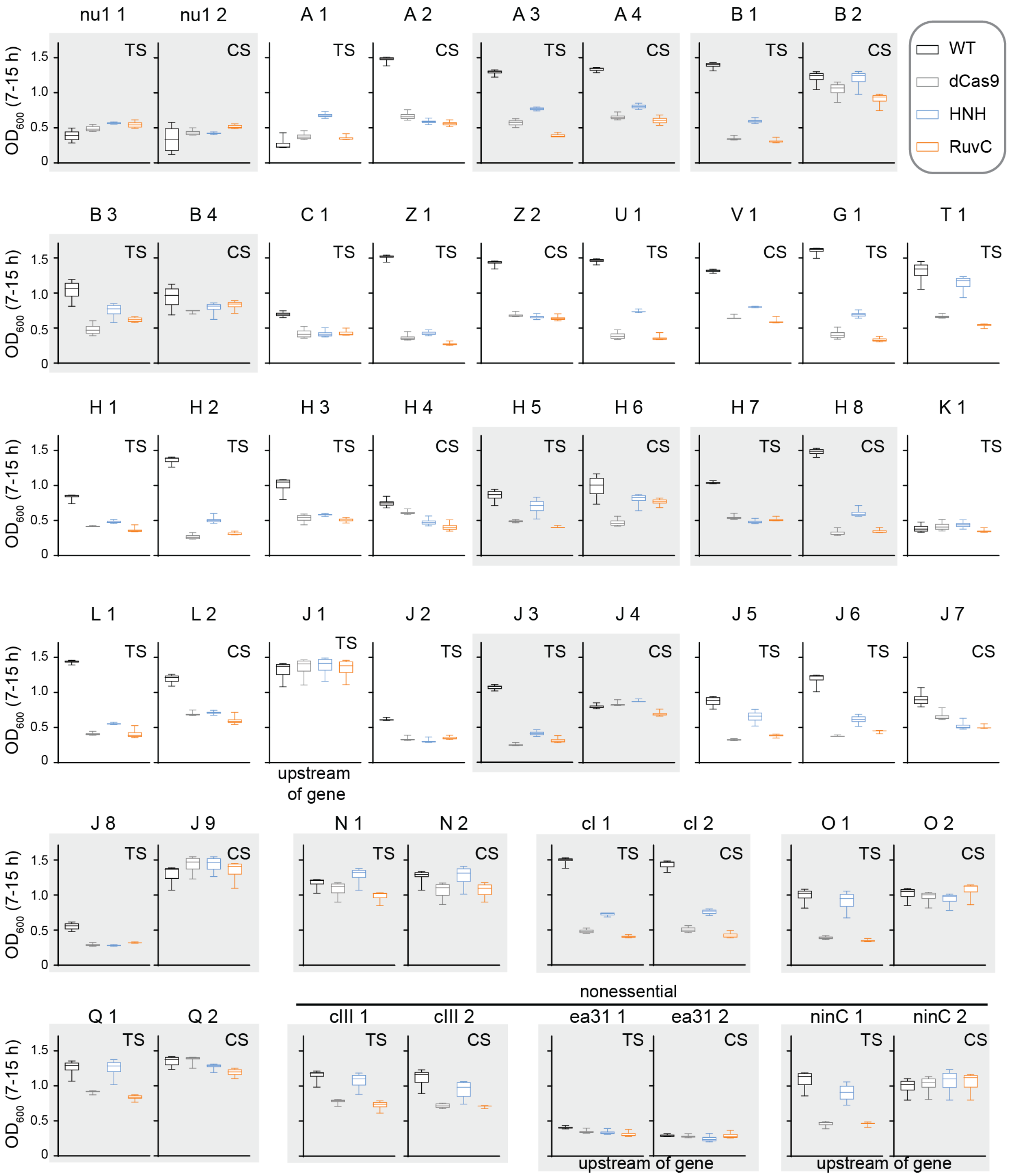

Figure S1: Protection by Cas9 variants for additional guide RNAs.

Box plots of OD600 values over the 7-15 h time period of growth curves, summarizing protection by four Cas9 variants using different guide RNAs. The gene targeted by the guide RNA is indicated. Plots that are outlined together in gray represent guide RNAs that target the

bioRxiv preprint doi: https://doi.org/10.1101/2024.09.05.611540; this version posted September 5, 2024. The copyright holder for this preprint (which was not certified by peer review) is the author/funder, who has granted bioRxiv a license to display the preprint in perpetuity. It is made available under aCC-BY 4.0 International license.

same or overlapping regions on either the template strand (TS) or coding strand (CS). Guide RNAs targeting non-essential genes or non-coding regions are indicated. All gene targets are provided in the Supplementary tables.

bioRxiv preprint doi: https://doi.org/10.1101/2024.09.05.611540; this version posted September 5, 2024. The copyright holder for this preprint (which was not certified by peer review) is the author/funder, who has granted bioRxiv a license to display the preprint in perpetuity. It is made available under aCC-BY 4.0 International license.

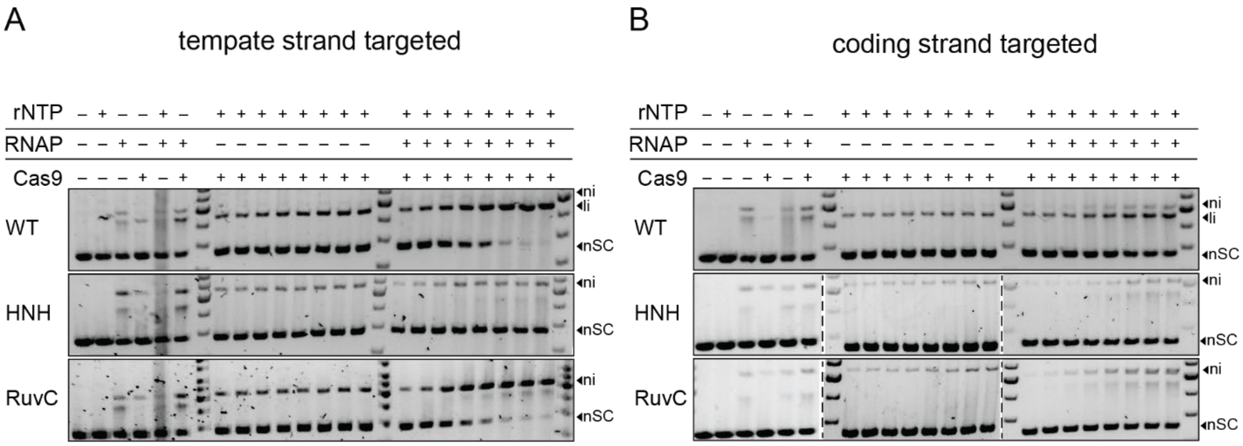

Figure S2: Gels showing Cas9 turnover by T7 RNA polymerase

Cleavage assays were performed with an excess of plasmid DNA over Cas9. In the absence of RNAP, only a fraction of the DNA is cleaved due to the lack of Cas9 turnover. When RNAP is added in the presence of rNTPs, Cas9 can turnover under some conditions tested, resulting in full cleavage of the DNA at longer time points.

A. The template strand of the target plasmid was targeted.

B. The coding strand of the target plasmid was targeted.

Supplementary Tables

List of λ genome targets

Primers for *cas9* mutagenesis and plasmids for Cas9 expression

qPCR primers

Oligonucleotides for crRNA synthesis and targets for cleavage assays
